# Comparación de las características epidemiológicas, clínicas y diagnósticas de endocarditis infecciosa de válvula nativa y protésica en un centro de referencia peruano

**DOI:** 10.47487/apcyccv.v6i1.463

**Published:** 2025-02-12

**Authors:** Daniel Espinoza-Alva, Renee Montesinos-Segura, Annette Mantilla-Huertas, Diego Davila-Flores

**Affiliations:** 1 Instituto Nacional Cardiovascular INCOR. Lima, Perú. Instituto Nacional Cardiovascular INCOR Lima Perú; 2 Instituto Nacional de Oftalmología, Lima, Perú. Instituto Nacional de Oftalmología Lima Perú

**Keywords:** Endocarditis, Diagnóstico, Signos y Síntomas, Perú, Endocarditis, Diagnosis, Signs and Symptoms, Peru

## Abstract

**Objetivo:**

. Comparar las características epidemiológicas, clínicas y diagnósticas de pacientes con endocarditis infecciosa de válvula nativa (EIVN) y protésica (EIVP) en un centro de referencia peruano.

**Materiales y métodos:**

. Estudio retrospectivo de pacientes con diagnóstico de EIVN y EIVP atendidos en el Instituto Nacional Cardiovascular (INCOR), EsSalud, entre 2017 y 2023. Se recolectaron datos clínicos y diagnósticos.

**Resultados.:**

Se incluyeron 65 casos de EIVN y 55 de EIVP, predominando la adquisición comunitaria (92,3 y 83,6%, respectivamente). La edad promedio fue mayor en EIVP (63,7 vs. 46,1 años, p<0,001), al igual que las comorbilidades. En EIVP, el factor predisponente más frecuente fue endocarditis previa (20,0%), mientras que en EIVN destacaron cardiopatías congénitas (41,5%) y valvulopatías (21,5%). Fiebre y disnea fueron síntomas comunes. La válvula aórtica fue la más afectada (78,5% en EIVN y 87,3% en EIVP). En EIVN predominaron vegetaciones (92,3%) y perforaciones (41,5%); en EIVP, abscesos (34,5%) y pseudoaneurismas (36,4%). La tomografía cardíaca permitió identificar vegetaciones y pseudoaneurismas en ambos grupos. Los hemocultivos fueron positivos en 49,2% de EIVN y 65,5% de EIVP, con predominio de estreptococos y estafilococos, respectivamente. El tratamiento quirúrgico se realizó en 96,7% de EIVN y 82,6% de EIVP.

**Conclusiones.:**

La EIVN predominó en jóvenes con cardiopatías congénitas, mientras que la EIVP afectó a pacientes mayores con comorbilidades. Los hallazgos microbiológicos y la ecocardiografía transesofágica fueron fundamentales para el diagnóstico.

## Abstract (en)

**Objective.:**

To compare the epidemiological, clinical and diagnostic characteristics of patients with native valve infective endocarditis (NVIE) and prosthetic valve infective endocarditis (PVIE) treated in a Peruvian reference center.

**Materials and Methods.:**

A retrospective, descriptive study was conducted on patients diagnosed with NVIE and PVIE at the Instituto Nacional Cardiovascular (INCOR), EsSalud, between 2017 and 2023.

**Results.:**

A total of 65 NVIE and 55 PVIE cases were identified, with community-acquired infections predominating (92.3% in NVIE, 83.6% in PVIE). PVIE patients were older (mean age: 63.7 vs. 46.1 years, p<0.001) and had more comorbidities. The key predisposing factor in PVIE was prior endocarditis (20.0%) and valve repair (14.5%), while congenital heart disease (41.5%) and valvular disease (21.5%) predominated in NVIE. Fever and dyspnea were the most common symptoms in both groups. The aortic valve was the most frequently affected (78.5% in NVIE, 87.3% in PVIE). NVIE was associated with vegetation (92.3%) and leaflet perforations (41.5%), while PVIE showed more abscesses (34.5%) and pseudoaneurysms (36.4%). Cardiac computed tomography identified vegetations and pseudoaneurysms in both groups. Blood cultures were positive in 49.2% of NVIE and 65.5% of PVIE, with Streptococcus species predominant in NVIE and Staphylococcus species in PVIE. Surgical treatment was performed in 96.7% of NVIE and 82.6% of PVIE cases.

**Conclusions.:**

NVIE predominantly affected younger patients with congenital heart disease, while PVIE was more common in older patients with comorbidities. Transesophageal echocardiography and microbiological findings were essential for diagnosis.

## Introducción

La endocarditis infecciosa (EI) es una enfermedad compleja caracterizada por la infección de la superficie endocárdica y las válvulas cardíacas ^1^^,^^2^. En la fisiopatología intervienen varios procesos, se inician con el daño endotelial debido al flujo turbulento, secundario a una cardiopatía estructural subyacente, formando trombos estériles; por lo que durante bacteriemias transitorias estos microorganismos se adhieren de forma directa o indirecta iniciando la infección ^1^^,^^2^. Los factores de riesgo para desarrollar EI incluyen factores cardiacos como: episodio previo de EI, portador de prótesis valvulares y dispositivos intracardiacos, cardiopatías congénitas y enfermedad valvular, así como factores no cardiacos como edad avanzada, sexo masculino, uso de drogas intravenosas, procedimientos dentales recientes, inmunosupresión y hemodiálisis ^3^^,^^4^.

La EI puede comprometer válvulas nativas (EIVN), válvulas protésicas (EIVP) y dispositivos electrónicos cardiacos implantables (DECI) siendo el 92%, 4% y 4%, respectivamente, de la población afectada ^5^^,^^6^. Para el diagnóstico de esta entidad se emplea una combinación de criterios microbiológicos, imágenes, quirúrgicos y clínicos; los criterios de Duke-ISCVID 2023 demostraron en estudios de validación externa, similar sensibilidad con mayor especificidad en comparación de criterios previos ^6^^,^^7^.

La epidemiología de esta entidad ha tenido considerables cambios en las últimas décadas con aumento en la incidencia global de 478 002 casos en 1990 a 1 090 526 en 2019; sin embargo, no se evidencian diferencias significativas en la mortalidad ni en la carga de enfermedad durante este periodo ^8^; por lo tanto, la identificación temprana y el tratamiento adecuado (médico o quirúrgico) son fundamentales para evitar complicaciones graves como embolización micromacrovascular, trastornos de conducción eléctrica, insuficiencia cardíaca, choque cardiogénico y la muerte ^3^^,^^8^. Dada la limitada disponibilidad de información sobre las características y tendencias de la endocarditis infecciosa (EI) en América Latina y el Caribe, resulta fundamental caracterizar y comparar a los pacientes con EI en válvulas nativas y protésicas para comprender mejor su diversidad clínica. En este contexto, el presente estudio tuvo como objetivo analizar las diferencias epidemiológicas, clínicas y diagnósticas entre ambas formas de EI en un centro de referencia peruano, con el fin de identificar factores predisponentes, manifestaciones clínicas y hallazgos microbiológicos e imagenológicos que contribuyan a optimizar su diagnóstico y tratamiento

## Materiales y métodos

### Diseño de estudio

Estudio observacional, descriptivo, retrospectivo en el Instituto Nacional Cardiovascular, INCOR-EsSalud, Lima, Perú, entre el 1 enero de 2017 al 31 de diciembre de 2023.

### Población

Se incluyó a todos los pacientes referidos a nuestro centro con el diagnóstico de endocarditis infecciosa (EI). Fueron separados en dos grupos de acuerdo con el tipo de válvula comprometida: válvula nativa o protésica (mecánica o biológica). Se utilizaron los criterios diagnósticos modificados Duke-ISCVID 2023^9^, los cuales describen I) Criterios patológicos; II) Criterios clínicos: mayores (microbiológico, imagen y quirúrgico) y criterios menores (predisposición, fiebre, fenómenos vasculares, fenómenos inmunológicos, microbiológico, imagen, examen físico). Se estableció el diagnóstico de EI definitiva si el paciente cumple uno de los siguientes criterios: a) Un criterio patológico; b) Dos criterios clínicos mayores; c) Un criterio clínico mayor con tres o cuatro criterios menores; d) Cinco criterios clínicos menores. El diagnóstico de EI posible se determinó si el paciente cumple uno de los siguientes: a) Un criterio clínico mayor con uno o dos menores; b) Tres o cuatro criterios clínicos menores.

### Variables

Se compararon ambos grupos en relación con factores de riesgo cardiovascular (edad, sexo, hipertensión arterial, diabetes *mellitus* tipo 2, tabaquismo, dislipidemia); antecedentes patológicos (enfermedad renal crónica, ictus, fibrilación auricular, cardiopatía isquémica, inmunosupresión); factores predisponentes, manifestaciones clínicas, hallazgos ecocardiográficos, hallazgos en la tomografía cardiaca, hemocultivos, microorganismo aislado, estudio de la válvula afectada, resultados histopatológicos y hallazgos quirúrgicos.

### Procedimientos o intervenciones

Para la recopilación de datos se revisaron las historias clínicas y se llenaron las fichas de recolección de datos correspondientes. Se excluyeron los casos con historias incompletas o casos considerados como recaída de endocarditis (un episodio de EI en los últimos 6 meses).

### Aspectos éticos

El estudio contó con la aprobación del comité de ética de la institución (047/2023 CEI). Debido a su diseño retrospectivo, no se requirió la obtención de consentimiento informado de los pacientes, ya que los datos fueron recolectados a partir de historias clínicas sin contacto directo con los participantes. Se garantizó la confidencialidad de la información, asegurando el cumplimiento de los principios éticos establecidos en la Declaración de Helsinki y en las normativas locales de investigación en salud. No se realizaron intervenciones que comprometieran la integridad o seguridad de los pacientes incluidos en el estudio.

### Análisis de datos

El procesamiento de datos se realizó con el *software* estadístico Jamovi versión 2.3.28.0. Las variables cualitativas se expresaron en frecuencias, y se compararon mediante la prueba de chi cuadrado o la prueba exacta de Fisher. Para las variables cuantitativas se calcularon la mediana o la media según su distribución, y se compararon mediante la prueba T de Student o prueba U de Mann-Whitney. Se consideró un valor de p < 0.05 como estadísticamente significativo. 

## Resultados

Se revisaron 131 historias clínicas, pero solo 120 pacientes fueron incluidos en el estudio, de los cuales 65 correspondían a casos de EIVN y 55 a EIVP. La adquisición comunitaria se observó en 60 casos (92,3%) de EIVN y en 46 casos (83,6%) de EIVP, mientras que la infección asociada a servicios de salud fue del 7,7% (5 casos) en EIVN y del 16,4% (9 casos) en EIVP.

Los pacientes con EIVP presentaron una edad promedio mayor y un mayor número de comorbilidades en comparación con aquellos con EIVN (Tabla 1). En cuanto a los factores predisponentes para EI, se encontró que el antecedente de EI previa fue más frecuente en la EIVP (20,0%), mientras que en la EIVN se observaron con mayor frecuencia cardiopatías congénitas (41,5%) y valvulopatías (21,5%). Entre las cardiopatías congénitas identificadas en la EIVN, se destacaron la válvula aórtica bicúspide (19 casos), la comunicación interventricular (3 casos), el conducto arterioso persistente (3 casos), la tetralogía de Fallot (1 caso) y la membrana subaórtica (1 caso). El factor predisponente como criterio menor estuvo presente en el 100% de los casos de EIVP y en el 53,8% de EIVN (Tabla 1).

**Tabla 1:** Factores de riesgo cardiovascular, antecedentes y factores predisponentes de los casos de endocarditis infecciosa de válvula nativa y protésica

	Nativas (%)	Protésicas (%)	Valor de p
Factores de riesgo cardiovascular			
Media de edad en años (DS)	46,1 (±17,8)	63,7 (±15,6)	<0,001
Sexo masculino	50 (76,9%)	37 (67,3%)	0,238
Hipertensión arterial	24 (36,9%)	30 (54,5%)	0,053
Diabetes	8 (12,3%	10 (18,2%)	0,369
Tabaco	12 (18,5%)	11 (20,0%)	0,831
Dislipidemia	6 (9,2%)	10 (18,2%)	0,151
Antecedentes			
Enfermedad renal crónica	6 (9,2%)	7 (12,7%)	0,539
Hemodiálisis	5 (7,7)	0 (0,0%)	0,062
Ictus previo	3 (4,6%)	9 (16,4%)	0,063
Cardiopatía isquémica	1 (1,5%)	10 (18,2%)	0,003
Fibrilación auricular	2 (3,1%)	13 (23,6%)	<0,001
Inmunosupresión	4 (6,2%)	1 (1,8%)	0,373
Procedimiento invasivo previo	8 (12,3%)	6 (10,9%)	0,812
Factores predisponentes			
Endocarditis infecciosa previa	0 (0,0%)	11 (20,0%)	<0,001
Portador de prótesis valvular quirúrgico o percutánea	1 (1,5%)	54 (98,2%)	<0,001
Reparación valvular quirúrgica o percutánea	0 (0,0%)	8 (14,5%)	0,001
Cardiopatía congénita	27 (41,5%)	2 (3,6%)	<0,001
Valvulopatía	14 (21,5%)	0 (0,0%)	<0,001
Portador de dispositivo electrónico intracardiaco	0 (0,0%)	1 (1,8%)	0,458
Cardiomiopatía hipertrófica	2 (3,1%)	0 (0,0%)	0,499
Uso de drogas endovenosas	1 (1,5%)	0 (0,0%)	1,000
Criterio menor por factor predisponente	35 (53,8%)	55 (100%)	<0,001

DS: Desviación estandar

El síntoma más común en ambos grupos fue la fiebre, aunque la disnea fue más frecuente en la EIVN (58,5%). El fenómeno vascular constituyó un criterio diagnóstico menor en el 36,9% de los casos de EIVN y en el 32,7% de los de EIVP, siendo la embolia arterial la manifestación más común. En cuanto a los fenómenos inmunológicos, su presencia fue poco frecuente en ambos grupos (Tabla 2).

**Tabla 2:** Manifestaciones clínicas, fenómenos vasculares y fenómenos inmunológicos de los casos de endocarditis infecciosa de válvula nativa y protésica

	Nativas (%)	Protésicas (%)	Valor de p
Manifestaciones clínicas			
Fiebre	52 (80,0%)	40 (72,7%)	0,348
Disnea	38 (58,5%)	20 (36,4%)	0,016
Síncope	3 (4,6%)	3 (5,5%)	1,000
Focalización neurológica	10 (15,4%)	9 (16,4%)	0,884
Fenómenos vasculares			
Embolia arterial	21 (32,3%)	16 (29,1%)	0,704
Infarto pulmonar séptico	3 (4,6%)	0 (0,0%)	0,249
Absceso cerebral o esplénico	1 (1,5%)	2 (3,6%)	0,593
Hemorragia intracraneal	2 (3,1%)	2 (3,6%)	1,000
Aneurisma micótico	0 (0,0%)	0 (0,0%)	
Hemorragia conjuntival	0 (0,0%)	0 (0,0%)	
Lesiones Janeway	2 (3,1%)	2 (3,6%)	1,000
Púrpura purulenta	0 (0,0%)	0 (0,0%)	
Hemorragias en astilla ungueales	0 (0,0%)	0 (0,0%)	
Criterio menor de fenómeno vascular	24 (36,9%)	18 (32,7%)	0,631
Fenómenos inmunológicos			
Factor reumatoideo	6 (9,2%)	5 (9,1%)	1,000
Manchas de Roth	0 (0,0%)	2 (3,6%)	0,208
Nódulo de Osler	2 (3,1%)	1 (1,8%)	1,000
Glomerulonefritis mediado por inmunocomplejos	1 (1,5%)	0 (0,0%)	1,000
Criterio menor por fenómeno inmunológico	9 (13,8%)	7 (12,7%)	0,857

Se realizó al menos un estudio ecocardiográfico en todos los pacientes, con una mayor frecuencia de ecocardiografía transesofágica en los casos de EIVP (98,2%). La válvula más afectada en ambos grupos fue la aórtica; sin embargo, la válvula mitral presentó un mayor compromiso en la EIVN en comparación con la EIVP. La afección multivalvular estuvo presente en el 33,8% de los casos de EIVN y en el 14,5% de EIVP. En el grupo de EIVP, la prótesis biológica fue la más afectada (74,5%). Con respecto a los hallazgos ecocardiográficos, en la EIVN se observaron con mayor frecuencia vegetaciones (92,3%), perforaciones de velo (41,5%) y nueva insuficiencia valvular (87,7%). En la EIVP se encontraron más abscesos (34,5%), pseudoaneurismas (36,4%) y dehiscencia parcial de prótesis (21,8%). La ecocardiografía constituyó un criterio mayor en el 100% de los casos de EIVN y en el 96,4% de los de EIVP (Tabla 3).

**Tabla 3:** Estudios de imagen cardiaca de los casos de endocarditis infecciosa de válvula nativa y protésica

	Nativas (%)	Protésicas (%)	Valor de p
Ecocardiograma	65 (100,0%)	55 (100,0%)	
Ecocardiografía transtorácica	63 (96,9%)	55 (100,0%)	0,190
Ecocardiograma transesofágica	58 (89,2%)	54 (98,2%)	0,05
Mediana de la FEVI en % (RIC)	60 (51-66)	60 (52-67)	0,825
Disfunción sistólica de ventrículo derecho	8 (12,3%)	6 (10,9%)	0,812
Válvula aórtica afectada	51 (78,5%)	48 (87,3%)	0,206
Válvula mitral afectada	30 (46,2%)	11 (20,0%)	0,003
Válvula tricúspide afectada	5 (7,7%)	2 (3,6%)	0,451
Válvula pulmonar afectada	4 (6,2%)	1 (1,8%)	0,373
Defecto congénito afectado	4 (6,2%)	0 (0,0%)	0,124
Vegetación	60 (92,3%)	35 (63,6%)	<0,001
Perforación de velo	27 (41,5%)	2 (3,6%)	<0,001
Aneurisma valvular	4 (6,2%)	0 (0,0%)	0,124
Absceso	7 (10,8%)	19 (34,5%)	0,002
Pseudoaneurisma	12 (18,5%)	20 (36,4%)	0,027
Fístula	9 (13,8%)	7 (12,7%)	1,000
Nueva insuficiencia valvular	57 (87,7%)	17 (30,9%)	<0,001
Dehiscencia parcial protésica	0 (0,0%)	12 (21,8%)	<0,001
Criterio mayor por ecocardiograma	65 (100,0%)	53 (96,4%)	0,208
Tomografía cardiaca	12 (18,5%)	20 (36,4%)	0,027
Vegetación	7 (58,3%)	4 (20,0%)	0,053
Perforación	1 (8,3%)	0 (0,0%)	0,375
Aneurisma valvular	1 (8,3%)	2 (10,0%)	1,000
Absceso	1 (8,3%)	5 (25,0%)	0,370
Pseudoaneurisma	2 (16,7%)	12 (60,0%)	0,028
Fístula	1 (8,3%)	2 (10,0%)	1,000
Dehiscencia protésica	0 (0,0%)	3 (15,0%)	0,274
Criterio mayor por tomografía	9 (75,0%)	16 (80,0%)	1,000
18-FDG PET CT	0 (0,0%)	3 (5,5%)	0,093
Criterio mayor por 18-FDG PET CT	0 (0,0%)	2 (66,7%)	
Criterio menor por 18-FDG PET CT	0 (0,0%)	1 (33,3%)	

*FEVI: fracción de eyección del ventrículo izquierdo. RIC: Rango intercuartílico.

** 18-FDG PET CT: tomografía por emisión de positrones con 18F-fluoro-2-desoxi-D-glucosa.

El uso de tomografía cardíaca como herramienta diagnóstica fue más frecuente en la EIVP (36,4%) que en la EIVN (18,5%). En los 12 pacientes con EIVN que fueron evaluados con tomografía, la vegetación fue el hallazgo más frecuente (18,5%). En los 20 pacientes con EIVP, los hallazgos predominantes fueron abscesos (25,0%) y pseudoaneurismas (60%). La válvula aórtica fue la más afectada en los estudios tomográficos en ambos grupos, con una prevalencia del 58,3% en EIVN y del 75,0% en EIVP (Tabla 3).

La tomografía por emisión de positrones (PET) se utilizó en solo tres pacientes con EIVP, en los cuales la indicación fue una alta sospecha clínica sin hallazgos concluyentes en ecocardiografía o tomografía cardíaca.

Se tomaron hemocultivos en todos los pacientes del estudio, siendo positivos en el 49,2% de los casos de EIVN y en el 65.5% de EIVP. Sin embargo, la mayoría de los pacientes había recibido antibioticoterapia antes del ingreso al centro y de la toma de hemocultivos. En la EIVN, los microorganismos más frecuentemente aislados fueron los estreptococos (24,6%), mientras que en la EIVP predominaron los estafilococos. El criterio mayor microbiológico fue más frecuente en la EIVP (43,6%) (Tabla 4).

**Tabla 4:** Resultados de hemocultivos de los casos de endocarditis infecciosa de válvula nativa y protésica

	Nativas (%)	Protésicas (%)	Valor de p
Hemocultivos	65 (100,0%)	55 (100,0%)	
Positivo	32 (49,2%)	36 (65,5%)	0,074
Criterio mayor microbiológico	9 (13,8%)	24 (43,6%)	<0,001
Criterio menor microbiológico	23 (35,4%)	12 (21,8%)	0,103
Microorganismo aislado			
Estafilococo aureus y coagulasa negativo	6 (9,2%)	17 (30,9%)	
Estreptococos orales y *Estreptococo gallolyticus*	16 (24,6%)	8 (14,5%)	
Enterococo	2 (3,1%)	8 (14,5%)	
Gramnegativo	1 (1,5%)	5 (9,1%)	
Cándida	1 (1,5%)	0 (0,0%)	
Otros	1 (1,5%)	0 (0,0%)	

Solo 79 pacientes del estudio tuvieron cultivos de válvula, prótesis valvular o material protésico, con resultados positivos en el 10,4% de los 48 casos de EIVN y en el 25,8% de los 31 casos de EIVP (Tabla 5). El estudio histopatológico se realizó en 53 pacientes con EIVN (58,9%) y en 28 con EIVP (60,9%), destacando que la presencia de endocarditis activa y el cumplimiento del criterio diagnóstico por patología fueron más frecuentes en la EIVN (Tabla 5).

**Tabla 5:** Resultados de cultivo de prótesis, estudios histopatológicos y hallazgos quirúrgicos de los casos de endocarditis infecciosa de válvula nativa y protésica

	Nativas (%)	Protésicas (%)	Valor de p
Cultivo de válvula, prótesis valvular o material protésico	48 (73,8%)	31 (56,4%)	0,044
Positivo	5 (10,4%)	8 (25,8%)	0,118
Estudio histopatológico	53 (86,9%)	28 (60,9%)	0,002
Microorganismo identificado	27 (50,9%)	12 (42,9%)	0,488
Endocarditis activa	48 (90,6%)	15 (53,6%)	<0,001
Criterio diagnóstico por patología	50 (94,3%)	20 (71,4%)	0,007
Tratamiento quirúrgico	61 (93,8%)	46 (83,6%)	0,085
Criterio mayor por hallazgo quirúrgico	59 (96,7%)	38 (82,6%)	0,018

En cuanto al tratamiento quirúrgico, 61 pacientes con EIVN fueron sometidos a cirugía, cumpliendo criterio mayor por hallazgo intraoperatorio en el 96,7% de los casos. En el grupo de EIVP, 46 pacientes recibieron tratamiento quirúrgico, de los cuales el 82,6% cumplieron criterio mayor diagnóstico quirúrgico (Tabla 5).

## Discusión

En el presente estudio se analizaron 120 casos de endocarditis infecciosa, clasificándolos en endocarditis en válvula nativa (EIVN) y protésica (EIVP). Los pacientes con EIVP eran mayores, con más comorbilidades y antecedentes de endocarditis, mientras que en EIVN predominaban las cardiopatías congénitas. Aunque la adquisición comunitaria fue común, la EIVP se asoció más con la atención en salud.

La fiebre fue el síntoma más frecuente, con disnea predominante en EIVN. Ecocardiográficamente, la EIVN presentó más vegetaciones y perforaciones, mientras que la EIVP mostró abscesos, pseudoaneurismas y dehiscencias protésicas. Los hemocultivos positivos fueron más frecuentes en EIVP, con predominio de estafilococos, mientras que en EIVN fueron más comunes los estreptococos.

El diagnóstico histopatológico y quirúrgico fue más habitual en EIVN, mientras que la tomografía cardíaca y la PET-CT, aunque menos utilizadas, identificaron pseudoaneurismas en EIVP. Estas diferencias reflejan patrones distintivos en epidemiología, clínica y diagnóstico.

Nuestros hallazgos muestran que los pacientes con EIVP tenían una edad promedio de 63,7 años, significativamente mayor que los 46,1 años en EIVN ^10^^,^^11^. Un estudio previo en Perú reportó una mediana de edad de 50 años, menor a la observada en nuestro análisis ^12^, lo que podría reflejar la tendencia global al aumento en la edad de presentación de la enfermedad en los últimos años. Este fenómeno se asocia con una mayor carga de comorbilidades en adultos mayores, como insuficiencia renal crónica, diabetes e hipertensión, factores que contribuyen a un peor pronóstico y mayor mortalidad ^12^^-^^14^. Además, la presentación clínica atípica y la menor frecuencia de fiebre dificultan el diagnóstico, aumentando el riesgo de complicaciones ^16^.

Etiologicamente tuvieron una frecuencia importante, el enterococo y el *Estreptococus gallolyticus*, este último vinculado a neoplasias colorrectales^10^^).^ Aunque la cirugía es la única opción curativa en muchos casos, su uso en ancianos es limitado por el alto riesgo quirúrgico; sin embargo, en pacientes seleccionados, mejora la supervivencia a largo plazo pese a mayores complicaciones posoperatorias ^(^^13^^,^^14^. Estos hallazgos destacan la necesidad de un enfoque multidisciplinario para optimizar el manejo y mejorar los desenlaces clínicos ^10^.

En cuanto al sexo, nuestros resultados muestran una ligera predominancia masculina en ambos grupos, en concordancia con registros de Europa y América Latina, como los de España y Argentina. En estos países, la endocarditis infecciosa es más frecuente en hombres, con una proporción varón-mujer que varía entre 2:1 y 3:1, independientemente de si se trata de EIVP o EIVN ^17^^-^^19^. Esta tendencia global podría explicarse por diferencias en los factores de riesgo predisponentes y en la exposición a procedimientos médicos entre géneros. Según el análisis de Slouha *et al*., la incidencia de EI es mayor en hombres, especialmente en válvulas nativas, mientras que las mujeres suelen desarrollar la enfermedad a edades más avanzadas, con predominio de la afección mitral y un mayor riesgo de complicaciones, como vegetaciones en dispositivos intracardiacos y válvulas protésicas ^20^. Además, las mujeres suelen recibir un manejo más conservador, lo que podría contribuir a la mayor mortalidad observada a los 30 días y al año en comparación con los hombres, quienes tienen mayor acceso a intervenciones quirúrgicas ^21^^).^. En Estados Unidos, los datos sugieren un aumento sostenido en la incidencia y mortalidad de la EI, con un incremento del 41,2% en la tasa de incidencia estandarizada por edad entre 1990 y 2019. Este crecimiento fue más pronunciado en hombres (45,8%) que en mujeres (34,1%) y afectó principalmente a personas mayores de 55 años, probablemente debido al envejecimiento poblacional y al mayor uso de dispositivos intracardiacos y procedimientos como el reemplazo valvular ^22^. Estos hallazgos resaltan la importancia de abordar las disparidades regionales, etarias y de sexo en el manejo de la EI, adaptando estrategias de diagnóstico y tratamiento a distintos contextos globales.

Nuestros resultados muestran que los pacientes con EIVP presentaron más comorbilidades cardiovasculares, como fibrilación auricular (23,6% vs. 3,1%, p<0,001) y cardiopatía isquémica (18,2% vs. 1,5%, p=0,003), en comparación con EIVN, lo que coincide con el registro europeo EURO-ENDO, que destaca la influencia de estas comorbilidades en el pronóstico y manejo ^3^.

Por otro lado, la EIVN se relacionó principalmente con cardiopatías congénitas, reflejando patrones descritos en Europa y América Latina ^3^. En adultos con cardiopatías congénitas, el riesgo de desarrollar EIVN es hasta 44 veces mayor que en la población general, debido a factores como *shunts* intracardiacos, alteraciones hemodinámicas y materiales protésicos, incluso tras correcciones quirúrgicas ^(^^23^^,^^24^. Condiciones como válvulas aórticas bicúspides, defectos septales ventriculares y tetralogía de Fallot son especialmente predisponentes, en particular tras procedimientos quirúrgicos o uso de dispositivos intracardiacos. Aunque la mortalidad intrahospitalaria es relativamente baja (4-9%), las complicaciones graves son frecuentes, lo que subraya la importancia de estrategias preventivas y un manejo multidisciplinario para optimizar el pronóstico en esta población ^24^^,^^25^.

En cuanto a las manifestaciones clínicas, la fiebre fue el síntoma más común en ambos grupos, mientras que la disnea predominó en la EIVN (58,5% vs. 36,4%, p=0,016), asociada probablemente con insuficiencia cardíaca secundaria a cardiopatías congénitas, como se ha reportado en Brasil ^26^. Además, diferencias clínicas y demográficas entre EIVN y EIVP resaltan la necesidad de un enfoque diagnóstico y terapéutico personalizado, considerando edad, comorbilidades y etiología, como sugiere una revisión reciente ^27^.Estas observaciones subrayan la relevancia de estrategias de manejo diferenciadas para optimizar los resultados.

Los hallazgos de este estudio coinciden con lo reportado en registros internacionales y destacan diferencias clínicas y diagnósticas entre la EIVN y la EIVP. En EIVN, la prevalencia de vegetaciones y perforaciones valvulares observada en este estudio es similar a la descrita en investigaciones realizadas en España y Colombia, donde estas lesiones, características de válvulas previamente sanas, se asocian con un mayor riesgo de embolización y deterioro hemodinámico ^(^^18^^,^^28^. Por otro lado, las complicaciones perivalvulares, como abscesos y pseudoaneurismas, predominan en EIVP y reflejan el impacto de la infección en válvulas protésicas, en concordancia con registros europeos que subrayan su adquisición predominantemente nosocomial, así como la mayor complejidad y gravedad de esta entidad clínica ^3^^,^^18^.

En nuestro estudio, los hemocultivos positivos fueron más frecuentes en EIVP (65,5%), predominando *Staphylococcus aureus* y estafilococos coagulasa-negativos, un hallazgo consistente con registros como el EURO-ENDO y el español, que asocian estos agentes etiológicos con infecciones relacionadas con servicios de salud y dispositivos intravasculares ^3^^,^^18^. En contraste, en la EIVN, los estreptococos fueron identificados en el 24,6% de los casos, reflejando una tendencia similar a la descrita en registros español, danés e italiano, donde los estreptococos están vinculados a infecciones comunitarias en válvulas previamente sanas ^17^^,^^18^^,^^29^. Estas diferencias microbiológicas subrayan la necesidad de adaptar el enfoque antimicrobiano según el tipo de válvula afectada y el contexto de adquisición de la infección. Asimismo, el predominio de *Staphylococcus aureus* en EIVP refleja los cambios globales en los factores predisponentes, como el aumento en la implantación de válvulas protésicas y la emergencia de resistencia antimicrobiana, lo que incrementa la complejidad del manejo clínico ^30^.

El uso extendido de la ecocardiografía transesofágica (ETE) en el 98,2 % de los casos de EIVP en este estudio subraya su papel esencial en la identificación de complicaciones como abscesos, pseudoaneurismas y dehiscencias protésicas, en concordancia con las recomendaciones internacionales^1^. Su elevada sensibilidad (90%-100%) en comparación con la ecocardiografía transtorácica (ETT) permite detectar con precisión lesiones graves que otros métodos podrían pasar por alto ^5^. De manera complementaria, la tomografía computarizada (TC) demostró ser crucial en casos complejos, particularmente para la identificación de abscesos, pseudoaneurismas y embolias sépticas, y fue utilizada principalmente en EIVP ^1^^,^^6^. Además, la combinación de PET-TC permitió detectar actividad inflamatoria en válvulas protésicas, consolidándose como una herramienta invaluable en centros de alta especialización(^30)^. Estas técnicas avanzadas fortalecen el diagnóstico integral de la EI, especialmente en escenarios complejos o de difícil evaluación.

Este trabajo tiene limitaciones por su diseño retrospectivo y el tamaño de la muestra, lo que podría subestimar ciertos hallazgos. Sin embargo, la inclusión de un análisis exhaustivo de factores clínicos, microbiológicos y de imagenología proporciona un valioso panorama de la endocarditis infecciosa en un país en desarrollo, permitiendo comparaciones significativas con registros internacionales.

Este estudio refuerza la necesidad de estrategias diferenciadas para el diagnóstico y manejo de EIVN y EIVP, subrayando la importancia de la vigilancia microbiológica y el uso temprano de modalidades avanzadas de imagenología. Investigaciones futuras deberían enfocarse en el impacto de intervenciones quirúrgicas y en el papel de herramientas diagnósticas como la PET-CT en entornos de recursos limitados.

En conclusión, este estudio proporciona información valiosa sobre las diferencias clínicas, microbiológicas y de manejo entre la EIVN y EIVP en un centro de referencia en Perú, destacando patrones epidemiológicos consistentes con registros internacionales. La EIVP estuvo asociada con mayor edad, comorbilidades y adquisición en servicios de salud, mientras que la EIVN predominó en pacientes más jóvenes con cardiopatías congénitas. La ETE demostró ser crucial en el diagnóstico, especialmente en casos de EIVP, mientras que los hemocultivos y el análisis histopatológico resaltaron las diferencias microbiológicas entre ambos grupos. Estos hallazgos subrayan la necesidad de un enfoque multidisciplinario para optimizar el diagnóstico temprano, el tratamiento oportuno y las estrategias preventivas, particularmente en entornos con recursos limitados; además, refuerzan la importancia de estudios futuros que exploren intervenciones quirúrgicas y herramientas avanzadas de imagen en este contexto.

## Fuente de financiamiento:

Autofinanciado
